# Problem-appropriate diagram instruction for improving mathematical word problem solving

**DOI:** 10.3389/fpsyg.2022.992625

**Published:** 2022-10-03

**Authors:** Hiroaki Ayabe, Emmanuel Manalo, Erica de Vries

**Affiliations:** ^1^Graduate School of Education, Kyoto University, Kyoto, Japan; ^2^Department of System Neuroscience, National Institute for Physiological Sciences, Okazaki, Japan; ^3^LaRAC, Univ. Grenoble Alpes, Grenoble, France

**Keywords:** self-constructed diagrams, instructional methods, mathematical word problem solving, cognitive load, representational effect, multiple baseline design, Japanese students, visual representation

## Abstract

The use of diagrams can be effective in solving mathematical word problems solving. However, students worldwide do not construct diagrams unprompted or have trouble using them. In the present study, the effects of problem-appropriate diagram use instruction were investigated with an adaptation of the multiple baseline design method. The instruction for using line diagrams, tables, and graphs was provided to 67 junior high school students in a staggered manner and the effects on problem solving of three different types of problems was examined. The results showed that use of problem-appropriate diagrams increased and persisted over time. More importantly, the instruction led to increases in problem solving performance and to decreases in perceived cognitive load. These findings support the argument that effective diagram use depends on the acquisition not only of declarative knowledge, but also sufficient procedural and conditional knowledge.

## Introduction

In mathematics education, teachers draw on mathematical word problem solving to facilitate application of acquired knowledge and skills to real and hypothetical problems and situations (Schoenfeld, 1985; Reed, 1999). Students, however, experience difficulties in solving word problems (Mayer et al., 1992; Reed, 1999; Jitendra et al., 2007; Boonen et al., 2014) since it requires more than simple retention and recall of facts and procedural steps. An effective heuristic to alleviate these difficulties is the use of diagrams (Hembree, 1992; Stylianou and Silver, 2004; Jitendra et al., 2007; Boonen et al., 2014). Diagrams facilitate self-explaining which in turn leads to deeper understanding (Ainsworth and Th Loizou, 2003), promote the construction of mental models for drawing inferences, and provide guidance towards appropriate learning behaviour (Butcher, 2006; van der Meij et al., 2017). More specifically in mathematics, diagrams enable the construction of accurate solutions by enhancing information and knowledge access (Chu et al., 2017; Cooper et al., 2018). However, although these studies contribute to understanding the role of diagrams in learning, they provide only limited insights about constructing effective diagrams for oneself. In many situations, students need to construct their own diagrams for solving word problems in classroom exercises, homework, or tests. Previous research shows several obstacles when students are required to construct their own diagram, instead of just inspect and manipulate a given diagram. Indeed, students may omit constructing a diagram, fail to construct an appropriate diagram, or still draw incorrect inferences from their diagram (Hegarty and Kozhevnikov, 1999; Uesaka and Manalo, 2006; Corter and Zahner, 2007; Uesaka et al., 2007; van Garderen et al., 2012). In the current study, we set out to study instruction as a way of improving students’ construction of appropriate diagrams in mathematical word problem solving.

### The representational effect or the appropriateness of a diagram for a specific problem

Diagrams enhance understanding of a problem through the representation of its elements and their interrelations (Hembree, 1992). In other words, they facilitate the construction of a schema or mental model of the problem text (Zahner and Corter, 2010). Solving a mathematical word problem involves two steps: generation of a problem representation from the text and implementation or computation of the solution (Kintsch and Greeno, 1985; Lewis and Mayer, 1987; Hegarty et al., 1995; Duval, 2006). The first step can be assimilated to a translation from one type of representation to another (Ainsworth, 2006), also termed conversion from one semiotic register to another (Duval, 2006). In Duval’s terminology, natural language, equations, and Cartesian graphs constitute different semiotic registers for representing abstract mathematical objects not directly available to the senses. For example, a problem stated in natural language can be converted into a line graph, but neither text nor graph can be equated with the mathematical object (e.g., a linear function) underlying the problem. Research shows the importance of this first step: when students construct an accurate visual-schematic representation of a problem situation, they are more likely to produce the correct answer (Hegarty and Kozhevnikov, 1999; Boonen et al., 2014).

Diagrams may also facilitate the second step of implementing the solution to the problem. Different isomorphic representations of the same abstract structure or mathematical object differ in their potential for solving a problem, termed “the representational effect” (Zhang and Norman, 1994; Zhang, 1997). Any problem can have multiple alternative forms of external representations (Schnotz and Kürschner, 2008). Thus, different types of diagrams attract attention to different features and may give “representational guidance” (Suthers, 2003). For example, tables attract attention to empty cells and may reveal patterns in a series of quantities in a problem. In mathematics, Duval spoke of “operational significance” (Duval, 2006): a representation in a particular semiotic register is meaningful because of the operations that it affords. For example, tables and graphs do not give a visual-schematic representation of the problem situation, but instead provide a schema for how the problem can be solved (Novick and Hurley, 2001; Zahner and Corter, 2010; Uesaka and Manalo, 2012). In effect, they facilitate what Duval (2006) described as transformations within the same semiotic register. For example, graphs allow visual inspection, which helps in identifying points of intersection of two or more trajectories.

There are a number of studies that have experimentally demonstrated the importance of matching problem requirements with representational affordances of diagrams. Hurley and Novick (2010), for example, asked participants to solve problems using diagrams that did or did not match the problem requirements. Predictably, they found poorer performance (i.e., longer time to solve, inaccurate inferences) in mismatched cases. It is clear therefore that in solving mathematical word problems, not just any diagram will be efficient: the kind of diagram selected and constructed must match the requirements of the problem at hand. We will call this *problem-appropriateness* of a diagram. Ideally, students need to acquire the whole repertoire of diagrams because problem solving in mathematics requires “representational flexibility” (Nistal et al., 2009) or “meta-representational competence” (diSessa, 2004; Verschaffel et al., 2020). In order to achieve meta-representational competence, students first need to acquire knowledge of the different types of representations. Grawemeyer and Cox (2008) demonstrated that such knowledge is crucial when solving “representationally specific tasks” (those that can only be carried out effectively with the use of a very limited range of representations).

Three kinds of representational knowledge are a prerequisite for effective diagram construction and use: declarative (knowing that), procedural (knowing how), and conditional (knowing when; cf. Paris et al., 1983; Garner, 1990). In sum, students need to know that certain kinds of diagrams are helpful for solving certain kinds of problems (declarative knowledge). They need to know how to correctly construct the appropriate diagram based on relevant information in the problem description (procedural knowledge). Finally, they need to know when to use a diagram as well as when to use a specific kind of diagram (conditional knowledge). The question arises whether instruction about representational knowledge of different types of diagrams would increase unprompted diagram use *per se*, and problem-appropriate diagram use in particular. In investigating different types of diagram instruction, the current study addresses this question and thus goes one step further than previous studies on the interplay between different types of diagrams and different types of problems.

### Cognitive load associated with constructing diagrams

One reason for the observed difficulties in constructing diagrams may lie in insufficient cognitive resources. From the perspective of cognitive load theory, problem solving tasks can only be successfully undertaken if the required or resulting cognitive load does not exceed the capacity of working memory (Sweller, 1994; Sweller et al., 1998). The effort for visually representing concrete details explicitly described in a word problem is low (e.g., illustrating details). In contrast, the construction of an abstract diagram that does not visually resemble the represented entities, such as a table or a graph, requires more transformational steps. Thus, such a construction is more difficult and demands higher amounts of cognitive effort (Uesaka and Manalo, 2012). Problem-appropriate diagram instruction may reduce cognitive load through schema construction (Sweller et al., 1998; Schnotz and Kürschner, 2007). Schemas cluster elements of a problem and its solution together making them more manageable. Problem-appropriate instruction may draw attention to specific problem features that provide clues for selecting the most appropriate diagram (Duval, 1999, 2006), as well as the relevant declarative, procedural, and conditional knowledge for actually constructing and using that diagram (cf. Paris et al., 1983).

### The present study

For developing knowledge about diagrams, appropriate instruction appears to be necessary (van Meter and Garner, 2005; Jitendra et al., 2007; van Garderen, 2007; Uesaka et al., 2010; Manalo et al., 2019). Although instruction appears to promote spontaneity in diagram use, the role of cognitive load and the effect on the correctness in problem solving, particularly where more complex problems are involved, has not been established. Our main purpose therefore was to investigate whether diagram instruction results in increases in *unprompted* diagram construction. Moreover, we expect an increase of *problem-appropriate* diagrams following corresponding diagram-specific instruction. As a result, correctness in solving corresponding word problems should increase and persist over time. Finally, we expect to see corresponding decreases in levels of perceived cognitive load when working on mathematical word problems.

Three diagram-specific instructions for line diagrams, tables, and graphs were designed and tested on three corresponding types of problems “Compare quantities,” “Predict patterns,” and “Compare trajectories” respectively. These types of problems and diagrams for solving them are a very important part of the Japanese school curriculum (Ayabe et al., 2021). An adaptation of the multiple baseline design method (Baer et al., 1968; Morgan and Morgan, 2009) was used in order to compare the use of different kinds of diagrams and performance on different types of problems across time following different types of instruction. This design involves giving the three types of instruction in a staggered manner and observing the effect on all types of problems for the same participants. For example, an increase in the use of line diagrams specifically and corresponding improvement in problem solving performance should only occur *after* the line diagram instruction and exclusively for the targeted Compare quantities problems, *not* the Predict patterns and Compare trajectories problems. Thus, this design is more appropriate than a “no instruction” control group because it allows comparisons of (1) the same students (within-participant design) and (2) several kinds of instruction. Our specific hypotheses were as follows:

*H1*: Diagram instruction leads to an overall increase in unprompted use of diagrams.

*H2*: Diagram instruction leads to an increase in the use of problem-appropriate diagrams persisting in time.

*H3*: Diagram instruction increases problem-solving performance (correct answer rates).

*H4*: Diagram instruction reduces perceived cognitive load.

## Materials and methods

A faculty ethics committee of Kyoto University approved the study. Participation was voluntary, and prior to the study, participants received verbal and written explanations. Informed consent was obtained from all participants and their parents.

### Participants

Seventy junior high school students (aged approximately 14 years, all Japanese) from three regular classes of a junior high school in a small city in Japan participated in the study (ability grouping is not usually practiced in schools in Japan). Students in Japan perform well in mathematics by world standards (Japan ranked 5th in mathematics in PISA 2018; OECD, 2019). We used G*Power (Faul et al., 2007) to estimate the minimum sample size for our within-participant design. This estimated that 46 participants would be required to detect a statistically significant difference for the assumed small to medium size effect (ƒ = 0.25, α-level *p* = 0.05, power = 0.80). Considering class sizes in the school (≤ 25 students), and allowing for dropout, three classes were included to ensure minimal sample size. The experimental sessions were conducted during regular class sessions. All the students participated but three missed some sessions and their data were excluded. Data from 67 students (female = 36) were used in the analyses.

### Problem-appropriate diagram instruction

Three dedicated instruction sessions covered the use of line, table, and graph diagrams. Instruction and practice sessions were held during regular class sessions (45 min duration). The instructions were given by the first author, assisted by a school mathematics teacher.

To ensure fidelity to plan and equivalence of the three instruction sessions, the authors discussed all contents and the instructional steps were determined in advance. PowerPoint slides were prepared and used to guide instruction. The instruction covered (1) the characteristics and functions of each kind of diagram (declarative knowledge), (2) the types and features of mathematics word problems that each diagram is useful for (conditional knowledge), and (3) the ways of constructing and the reasoning behind each diagram (procedural knowledge). During practice, the students solved example problems and constructed diagrams individually.

#### Line diagram instruction

Line diagrams, also known as “line numbers” or “tape diagrams” (Murata, 2008), visually express quantities as line segments. Line diagrams allow inferring relationships between sums, differences, multiples, and proportions (declarative knowledge). Constructing a line diagram involves converting quantities to lines to enable easier visual comparisons of the lengths of the lines. Conditional knowledge included that line diagrams are helpful for solving complex problems about relationships between quantities. For developing procedural knowledge, students were asked to construct line diagrams in solving three word problems (isomorphic but different from those used in the tests).

#### Table instruction

The instructor explained and demonstrated how tables are effective for organizing numbers or quantities of two variables of interest. The students were told that creating an array for one variable and then arranging the second variable in a corresponding array would clarify the relationship between the two variables. Thus, a table makes it easier to find the rule that determines how the two variables change (declarative knowledge). The conditional knowledge conveyed was that tables are helpful for identifying a consistent pattern or rule of change in quantities to predict a future amount. For developing procedural knowledge, the students practiced constructing tables for use in solving three isomorphic word problems.

#### Graph instruction

The instructor explained that graphs (more specifically, cartesian graphs) are useful for visually representing complex variations or changes of quantities and gave a demonstration on how to represent two variables of a word problem as points with connecting lines on the x-and y-axes. The declarative knowledge included that graphs enable visual awareness of the change in quantities as they increase, decrease, or remain the same across space and time. It also included knowledge about how graphs can be used, such as extending two lines on graphs to find their intersection. The conditional knowledge conveyed was that a graph should be used for complicated processes of change that require projections of future events. Again, for developing procedural knowledge, the students practiced constructing graphs in solving three isomorphic problems.

### Mathematical word problems

Five isomorphic problems (same problem structure but with different cover stories) for each of three problem types (Compare quantities, Predict patterns, and Compare trajectories) were used for three types of problem-appropriate diagrams (line, table, and graph diagrams respectively).

Compare quantities problems contained information about the magnitudes of lengths or distances. Solving these problems involved comparing these quantities. Line diagrams are appropriate because constructing a correct visual representation of the lengths not only provides a schematic layout of the problem situation, but also supports identification and working out of missing or unknown lengths (see example problems in Table 1).

**Table 1:** Example problems (translated from Japanese) and student-constructed problem-appropriate diagrams.

**Compare quantities problem**	**Line**
There are three counters A, B, and C, at a concert venue for customers with A, B, and C-type tickets. Upon opening, queue length at counter A is 72 m, unknown at counter B, and 56 m at counter C. Fifteen minutes after opening, the queue length at A is 6 m shorter than twice the length at B, and the length of C is 1 m shorter than half the length at A. How much did queue length shorten in the first 15 min? How long was the queue length at B when the gate was opened? (counters become shorter at constant and identical speed).	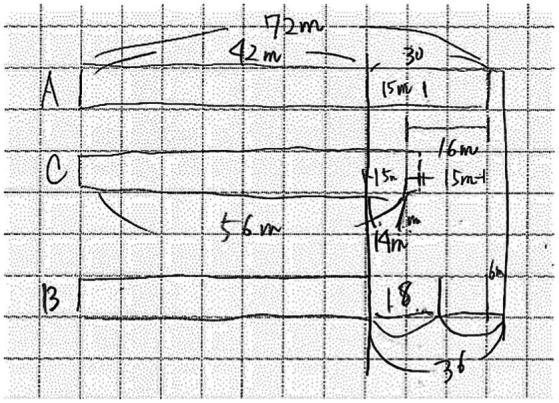
**Predict patterns problem**	**Table**
You have to arrange regular hexagonal tiles of 3 cm sides one at a time. Each new tile has to touch only one side of the tiles that are already placed. However, once placed, a tile can have more than one of its sides touching other tiles. When the number of sides (sides not in contact with other sides) around the figure becomes 86, how many tiles will you have arranged? When 26 tiles are placed, what is the length (in cm) around the figure?	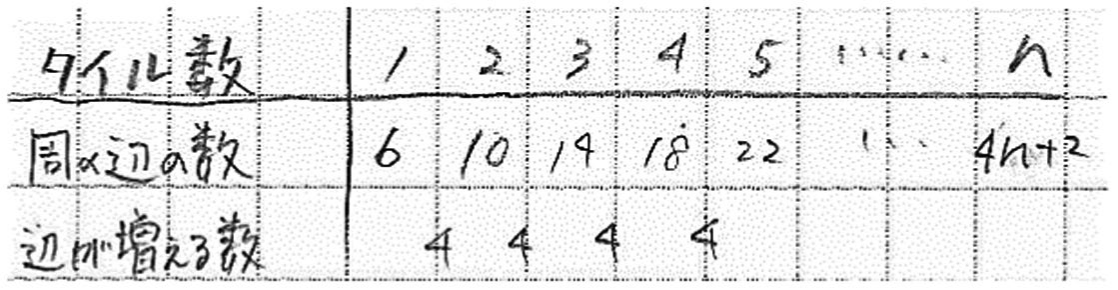
**Compare trajectories problem**	**Graph**
Manny leaves home at 6:30 am and walks 1,650 m to school. At school, he discovers that he forgot his lunch box and goes back. His mother discovers the lunch box and decides to bring it to him. At 7:18 am, Manny calls his mother’s mobile phone from a convenience store 900 m away from his school. She tells him that she already passed the convenience store at 7:10 am. They meet at the convenience store and Manny gets his lunch box. What time did his mother leave home? How long did Manny stay at school before returning home? (Manny and his mother walk at the same speed. The house, convenience store, and school are on the same route.)	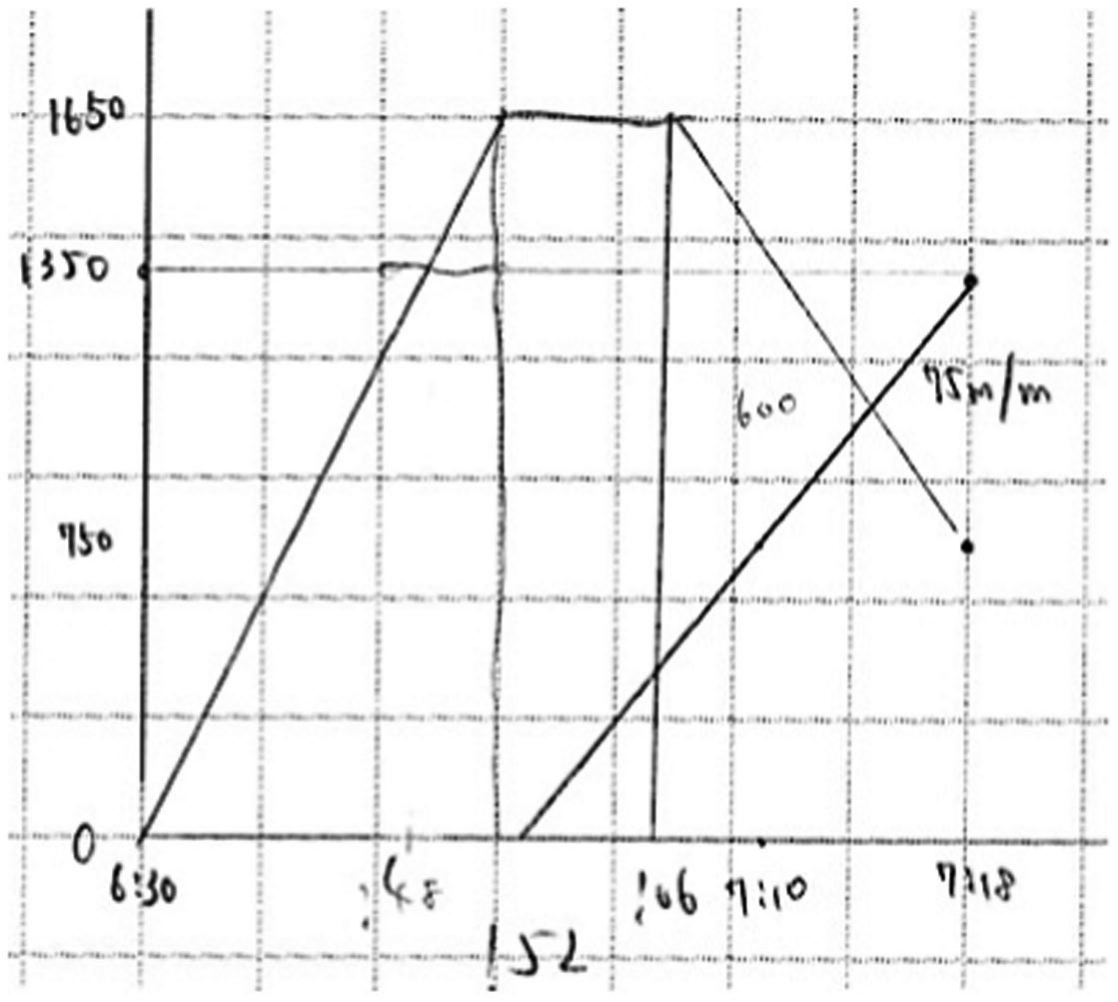

Predict patterns problems contained information about quantities at multiple times or stages. Students were not informed of the rule-based character of the changes. Solving these problems required students to infer the rule and predict future quantities. Tables are appropriate because their structure makes patterns of changes visible, which leads to apprehending the underlying rule.

Compare trajectories problems contained information about actions from two or more entities (usually people). Solving these problems required students to compare trajectories of the different entities. Graphs are appropriate because they enable plotting distances (relative to a point of reference) across time, which in turn enables comparing trajectories.

Prior to the study, the 15 problems (5*3) were given to five mathematics teachers (female = 1; mean teaching experience = 9.2 years, *SD* = 2.8 years) to check whether they were comparable and suitable for the intended grade level (14-year-olds at junior high school). Minor adjustments were made based on the teacher feedback. The revised problems were administered to 29 students from another school (female = 12; mean age = 13.2 years). Multiple comparisons using paired t-tests of the correct answer rates revealed no significant differences between the five problems of each type. Thus, they were considered equivalent and randomly used in the five test phases: Pre-test, Post-test after each of the three instruction sessions, and Delayed post-test.

### Dependent measures

#### Unprompted and problem-appropriate diagram use

An analysis grid was constructed for scoring the kind of diagram (Table 2). Numbers, equations, formulas, or computations in columns were not considered as diagrams.

**Table 2:** Analysis grid for scoring constructed diagrams.

Line	A line diagram consists of line segments or rectangular forms (tapes, bars) representing quantities. Two or more segments should be aligned so that their lengths can be compared. Segments without units or scales such as in geometric shapes or pictures should not be counted as a line diagram.
Table	A table contains at least two arrays of numbers resulting in a matrix of at least two by three (2 × 3) cells. A table need not have a legend, labels, or borders. A 1×2 or 2 × 2 table or an incomplete table (< 6 cells) should not be counted as a table.
Graph	A graph is a Cartesian coordinate system for plotting at least two functions in which a quantity (cost, distance) varies in time. The points and lines do not need to be correct. An empty x-y plane without points or lines is not counted as a graph.
Illustration	Any other graphical or pictorial visual expression or depiction.

Two teachers, with no vested interest in the study, rated all 1,005 answer sheets (5*3*67) in random order, blind to both test phase (5 phases) and type of problem (3 types). The teachers first rated 20% of the answer sheets, and compared and discussed their ratings with the first author. The teachers then independently scored the remaining answer sheets. Overall interrater agreement was high (Cohen’s kappa = 0.918). Unprompted diagram use was calculated as the presence of any kind of diagram. Problem-appropriate diagram use was calculated as the use of a specific kind of diagram for a specific type of problem (line diagram for Compare quantities, table for Predict patterns, and graph for Compare trajectories problems).

#### Correctness in problem solving

Correctness in problem solving was scored independently of diagram use. For each question (answer sheet), two answers were required for 0.5 points each. Correctness was scored 1 if both answers were correct, 0.5 if only one of them was correct, and 0 if both were incorrect or answers were missing.

#### Cognitive load

Cognitive load was measured using a short questionnaire for intrinsic cognitive load (Leppink et al., 2014) translated to Japanese, with some minor adjustments. The questionnaire comprised four items, for example “I invested a very high mental effort in the complexity of this activity,” to be answered on a 10-point Likert-type scale (0 = “not at all the case” to 9 = “completely the case”). The reliability of the scale was confirmed on the 15 problems in the preliminary study (Cronbach’s alpha ranged from 0.67 to 0.93).

### Design and procedure

Following the multiple baseline method, instruction in the use of line diagrams, tables, and graphs was provided in a staggered manner in three sessions, respectively, (see Table 3).

**Table 3:** Summary of the multiple baseline design. All five test phases contained a Compare quantities, a Predict patterns, and a Compare trajectories problem.

Session	Day	Instruction	Test Phase
1	1		Pre-test
2	6	Line	Post line test
3	9	Table	Post table test
4	13	Graph	Post graph test
5	22		Delayed test

The procedures used in administering the tests were identical across the five phases. Each test contained the three types of word problems in random order. Students were given 8 min to solve each problem. Students filled out the cognitive load questionnaire after solving each problem. All answer sheets were collected at the end of each session. No marks, grades, or feedback on the tests were given in between sessions.

### Analyses

Unprompted and problem-appropriate diagram use were dichotomous dependent variables (0 or 1). Therefore, Cochran’s Q, a non-parametric test, was used for analysis of main phase effects and McNemar’s test was used for pairwise comparisons. Correctness in problem solving had three possible scores (0, 0.5, 1) and perceived cognitive load ranged from 0 to 36. A repeated-measures analysis of variance was run on these variables as it is robust against violations of normal distribution assumptions (Schmider et al., 2010). The Greenhouse–Geisser correction was used when the sphericity assumption was not met. We performed confirmatory analysis with the non-parametric Friedman test.

## Results

### Did diagram instruction lead to an overall increase in unprompted use of diagrams?

**Figure 1:**
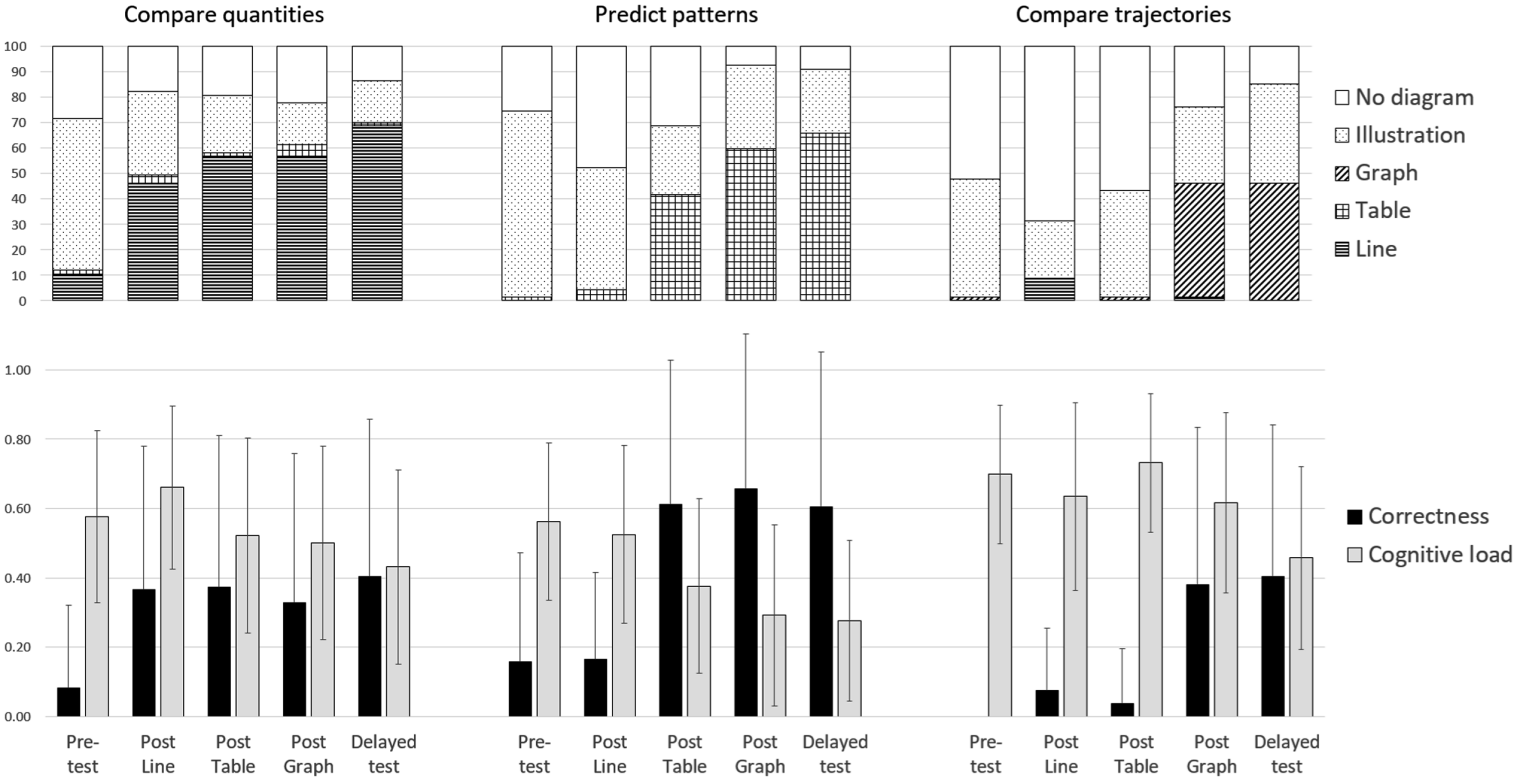
Diagram use **(top)**, correctness in problem solving and perceived cognitive load **(bottom)** as a function of problem type and test phase. Cognitive load was normalized to range from 0 to 1.

Figure 1 shows diagram use (top row) as a function of problem type and test phase and allows comparing the percentage of answer sheets that included a diagram of any of the four kinds (cumulated shaded parts of the bars) against those that did not include any diagrams at all (white part of the bars). As expected, the unprompted use of any diagram seems to increase as a result of the instructions (white portion decreases over time) but only for the Predict patterns and Compare trajectories problems.

The analysis showed no significant phase effect for Compare quantities problems [Cochran’s *Q*_(4)_ = 7.26, *p* = 0.12]. Moreover, no significant difference was found in diagram use between the tests immediately before and after the line instruction [Pre-test versus Post line, McNemar’s *χ*^2^_(1)_ = 2.88, *p* = 0.90, *p* values were multiplied by 10 as a Bonferroni correction]. Thus, the overall level of unprompted diagram use of any kind of diagram was high for Compare quantities problems (> 70%) from the beginning and stayed at such a high level throughout the five phases.

In the Predict patterns problems, a significant phase effect was found (Cochran’s *Q*_(4)_ = 48.35, *p* < 0.001). However, no significant difference was found in unprompted use of any diagram between the tests immediately before and after the table instruction [Post line versus Post table McNemar’s *χ*^2^_(1)_ = 4.84, *p* = 0.28]. A significant increase was observed only after graph instruction [Post line versus Post graph McNemar’s *χ*^2^_(1)_ = 22.09, *p* < 0.001]. Moreover, unprompted use of any diagram for Predict patterns problems did not increase nor decrease from the Post graph to the Delayed test [Post graph versus Delayed McNemar’s *χ*^2^_(1)_ = 0.11, *p* = 1.00]. Thus, unprompted use of any diagram increased following table instruction in the corresponding Predict patterns problems, but only towards the end of the procedure.

Finally, a significant phase effect was also found for Compare trajectories problems [*Q*_(4)_ = 64.55, *p* < 0.001]. The significant increase in unprompted use of any diagram followed graph instruction [Post table versus Post graph McNemar’s *χ*^2^_(1)_ = 13.44, *p* < 0.01]. It seemed to still increase from the Post graph to the Delayed test, but this was not significant [Post graph versus Delayed McNemar’s *χ*^2^_(1)_ = 3.00, *p* = 0.83]. Hence, following graph instruction, unprompted diagram use of any diagram for the corresponding Compare trajectories problems significantly increased and sustained.

### Did diagram instruction lead to a persisting increase in problem-appropriate diagrams?

We expected an increase of problem-appropriate diagrams specifically. In other words, we expected increases in the use of line diagrams for comparing quantities, tables for predicting patterns, and graphs for comparing trajectories. Such problem-appropriate diagram use should occur directly following the corresponding instruction and persist in time even after alternative diagram instruction. Figure 1 does show this expected pattern of results. The use of each of the three types of diagrams increases after the corresponding instruction but only for the expected type of problem. Following line instruction, although some line diagrams were also used for comparing trajectories, the use of line diagrams increased for comparing quantities [phase effect in line diagram use, Cochran’s *Q*_(4)_ = 69.30, *p* < 0.001], but not for the other two types of problems. Following table instruction, the use of tables increased for predicting patterns [phase effect in table use, *Q*_(4)_ = 121.39, *p* < 0.001], not for the other two types of problems. And finally, following the graph instruction, the use of graphs increased for comparing trajectories [phase effect in graph use, *Q*_(4)_ = 105.86, *p* < 0.001], again not for the other two types of problems.

Individual comparisons (again with Bonferroni corrections) confirmed the pattern of results. In the Compare quantities problems, the increase in line diagram use took place directly after the line instruction [between Pre-test and Post line test, McNemar’s *χ*^2^_(1)_ = 19.20, *p* < 0.001]. It seemed to still increase in the Delayed test. Indeed, there was a significant difference between the Post line and Delayed test [*χ*^2^_(1)_ = 10.71, *p* < 0.01]. Thus, as Figure 1 shows, appropriate use of line diagrams for Compare quantities problems directly followed line instruction and still increased even after alternative table and graph instructions.

Similar results were obtained for the Predict patterns problems. Following table instruction, table use increased significantly for solving these problems [between Pre-test to Post table test, *χ*^2^_(1)_ = 27.00, *p* < 0.001] and increased still further from Post table to the Delayed test [*χ*^2^_(1)_ = 16.00, *p* < 0.001]. Thus, appropriate use of tables for Predict pattern problems started directly after instruction, and even continued to increase, rather than decrease, after alternative diagram instruction.

Finally, in the Compare trajectories problems, graph use increased significantly just after the graph instruction [between Pre-test to Post graph test, *χ*^2^_(1)_ = 29.00, *p* < 0.001]. However, unlike above, appropriate diagram use did not further increase in the Delayed test.

In all cases, the increase in problem-appropriate diagrams was observed directly after instruction but exclusively for the corresponding type of problem. Moreover, problem-appropriate diagram use tended to intensify, even despite the instruction on alternative diagrams. These results provide full support for the second hypothesis.

### Did diagram instruction increase correctness in problem solving?

We expected an increase in correctness for each problem type directly following the corresponding diagram instruction and remaining stable over time. Such an improvement in problem solving can indeed be seen in Figure 1 (black bars in bottom bar graphs). Since the diagram-appropriate instruction took place in a staged fashion, the number of baseline data points differs for the three problem types, Comparing quantities, Predicting patterns, and Comparing trajectories (one, two, and three baseline data points, respectively). We therefore tested the pattern of results with three separate repeated-measurements analysis of variance, one for each problem type.

ANOVA revealed a significant phase effect for Compare quantities problems, *F*_(4, 264)_ = 9.24, *p* < 0.001, η_G_^2^ = 0.08. Comparing adjacent phases showed that only the first contrast, between Pre-test and Post line test, reached significance, *t*_(66)_ = 5.11, *p* < 0.001, Cohen’s *d* = 0.88, all *p* values were Bonferroni adjusted. Thus, correctness for Compare quantities problems significantly increased following line diagram instruction and the higher level of performance in problem solving persisted throughout the four subsequent test phases.

We also found a significant test phase effect for Predict patterns problems [*F*_(3.34, 220.12)_ = 40.06, *p* < 0.001, η_G_^2^ = 0.26]. For this type of problem, Figure 1 shows that correctness increased directly after the appropriate table instruction. Indeed, in comparing adjacent test phases, the contrast for the comparison between Post line and Post table tests was significant, *t*_(66)_ = 8.95, *p* < 0.001, *d* = 1.55. Thus, correctness for Predict pattern problems augmented after the table instruction and the higher level maintained throughout the subsequent tests.

Finally, the test phase effect was significant for Compare trajectories problems, *F*_(2.39, 157.56)_ = 33.03, *p* < 0.001, η_G_^2^ = 0.26. Figure 1 clearly shows improved problem solving directly after the problem-appropriate graph instruction. This expected distinct increase in correctness after graph instruction was significant, *t*_(66)_ = 6.31, *p* < 0.001, *d* = 1.09. Improved correctness for Compare trajectories problems sustained at the obtained higher level in the Delayed test.

Friedman test results provided confirmation of these significant results in the Compare quantities problems [*χ*^2^_(4)_ = 34.15, *p* < 0.001], the Predict patterns problems [*χ*^2^_(4)_ = 99.04, *p* < 0.001], and the Compare trajectories problems [*χ*^2^_(4)_ = 90.93, *p* < 0.001]

Finally, we examined the relation between the use of problem-appropriate diagrams and correctness in problem solving in the Delayed test. Chi-square tests for contingency tables showed that the students who produced an appropriate diagram also obtained higher correctness in problem solving [Compare quantities, *χ*^2^_(2)_ = 7.16, *p* < 0.05; Predict patterns, *χ*^2^_(2)_ = 19.30, *p* < 0.001; Compare trajectories, *χ*^2^_(2)_ = 12.83, *p* < 0.01]. These results show that the use of problem-appropriate diagrams is indeed concurrent with correctness in problem solving, providing full support for the third hypothesis.

### Did diagram instruction reduce perceived cognitive load?

Finally, we expected that diagram instruction would decrease perceived cognitive load. Figure 1 shows that while the perceived cognitive load seems slightly decreasing over time (gray bars in bottom bar chart), the relation to diagram instruction is less marked. Again, we ran a separate analysis for each of the three problem types for the same reason given above.

The ANOVA showed a significant phase effect in the Compare quantities problems [*F*_(3.52, 232.01)_ = 14.51, *p* < 0.001, η_G_^2^ = 0.08]. Unexpectedly, perceived cognitive load actually increased significantly following line diagram instruction [Pre-test versus Post line test, *t*_(66)_ = 2.81, *p* < 0.05, *d* = 0.49]. Subsequently, a significant decrease took place from the Post line to the Post table test, *t*_(66)_ = 4.14, *p* < 0.001, *d* = 0.72. Perceived cognitive load was lowest at Delayed test (significantly lower than at Pre-test, *t*_(66)_ = 4.13, *p* < 0.001, *d* = 0.71). In other words, line diagram instruction did not immediately lead to cognitive load reduction in solving the Compare quantities problems, but a delayed reduction could be observed.

A significant phase effect was also found for the Predict patterns problems, *F*_(3.58, 236.09)_ = 35.78, *p* < 0.001, η_G_^2^ = 0.19. In the Predict patterns problems, the pattern of perceived cognitive load variations fully supported the fourth hypothesis. No change in reported cognitive load was found prior to table instruction [Pre-test versus Post line test, *t*_(66)_ = 1.21, *p* = 0.46 (ns), *d* = 0.21], but a significant decrease followed table instruction [*t*_(66)_ = 4.37, *p* < 0.001, *d* = 0.76, as well as a further decrease observed in the next Post graph test, *t*_(66)_ = 2.95, *p* < 0.05, *d* = 0.51. Cognitive load did not further decline in the Delayed test, *t*_(66)_ = 0.64, *p* = 0.52 (ns), *d* = 0.11]. Thus, evidence was found that table instruction reduced perceived cognitive load in solving the corresponding Predict patterns problems.

Finally, the analysis of perceived cognitive load showed a main effect of test phase for the Compare trajectories problems, *F*_(3.41, 224.80)_ = 22.77, *p* < 0.001, η_G_^2^ = 0.14. The contrasts showed that, while there was no change in perceived cognitive load between Pre-test and Post line test, there was an unexpected increase at Post table test [i.e., Post line test versus Post table test, *t*_(66)_ = 3.70, *p* < 0.01, *d* = 0.64]. Following graph instruction, the reported load then significantly decreased [i.e., Post table test versus Post graph test, *t*_(66)_ = 3.38, *p* < 0.01, *d* = 0.58]. A further decrease in perceived cognitive load was found at the Delayed test [Post graph test versus Delayed test, *t*_(66)_ = 5.50, *p* < 0.001, *d* = 0.95]. It is possible to interpret the decline in cognitive load from Post table test through to Delayed test as possibly stemming from practice effects. However, given that no decrease in perceived cognitive load actually occurred until *after graph instruction was provided,* we believe that on the whole these results can be taken as supporting the fourth hypothesis.

## Discussion

The results of the present study provide support for the hypotheses that we tested. Diagram instruction increased unprompted use of diagrams and, more importantly, it increased the use of problem-appropriate diagrams. These increases in use persisted in time. Furthermore, the instruction led to increases in student problem solving performance and to decreases in their perception of cognitive load associated with that problem solving. In this section, we consider the reasons for and meaning of these results, and discuss their theoretical, research, and practical implication.

### Promoting unprompted and problem-appropriate diagram use

Two previously identified key challenges are that students generally lack spontaneity in diagram use and that, even when they construct diagrams, these are often not appropriate for the problem (Hegarty and Kozhevnikov, 1999; Uesaka and Manalo, 2006; Corter and Zahner, 2007; Uesaka et al., 2007; van Garderen et al., 2012). The findings of the present study demonstrate that with instruction focusing on the correspondence between different types of problems and different kinds of diagrams both of these challenges can be resolved.

Previous research revealed that deficiencies in declarative knowledge is one important reason why students do not use diagrams when they should. Previous research also showed that instruction promotes greater spontaneity in the use of diagrams in mathematical word problem solving (Uesaka et al., 2010). This was confirmed in the present study: there were significant increases in unprompted use of any diagrams in both the Predict patterns and Compare trajectories problems following diagram instruction. In the Compare quantities problems, increases in the unprompted use of any diagrams also followed instruction, but these were not significant. The most likely reason was that the level of diagram use in attempts at solving the Compare quantities problems was already high at the first baseline (Pre-test), and so the increases that followed were proportionally small. Note that in all three problem types, most of the diagrams that participants constructed prior to instruction were illustrations (see Figure 1), which would not have been helpful toward obtaining the correct solutions.

Instruction specifically should promote the construction of effective diagrams. Thus, in the present case, the goal of instruction was not for students to construct any diagram because not all diagrams are equal in helping toward generating the required answers. Different representations, even when they are isomorphic, vary in their potential for solving a problem (Zhang and Norman, 1994; Zhang, 1997; Duval, 2006; Schnotz and Kürschner, 2008). Therefore, a crucial purpose of instruction is to enable students to determine and construct the most appropriate diagram to match the requirements of a problem. In the present study, for all three problem types, significant increases in problem-appropriate diagrams were evidenced following instruction, and those increases maintained. In the test phases following each instruction session, all three problem types were administered (in a random order) but, in each case, a significant increase was observed only in the problem type corresponding to the kind of diagram for which instruction had just been provided. This result suggests that, when given instruction, students are able to distinguish pertinent features of a problem and consequently select the most appropriate kind of diagram for solving it. They are able to develop both the necessary conditional and procedural knowledge.

### Reducing cognitive load and improving word problem solving

A third important point is that, if students construct a problem-appropriate diagram, it should lead to a better problem solving performance. Again, this was demonstrated in the present study: the increases in appropriate diagram use coincided with significant improvements in problem solving performance. This outcome is understandable when we consider the representational effect mentioned earlier (Zhang and Norman, 1994; Zhang, 1997) and the specific operations that representations can enable in mathematical problem solving (Duval, 2006). More specifically, while some diagrams give an accurate visual-schematic representation for understanding a problem (Hegarty and Kozhevnikov, 1999; Boonen et al., 2014), they may not help in actually solving it. Problem-appropriate diagrams, especially for more complex mathematical word problems, are not just visual or topographical representations. They are of a more abstract nature that enables drawing inferences or executing necessary operations. The execution of such operations is quite specific and systematic, requiring the connections between pertinent details in the problem text, the choice and construction of the diagram, and the derivation of the solution, to be explicitly explained – and practiced – in instruction sessions provided.

This brings up a fourth important point: that problem-appropriate instruction likely reduces the cognitive load experienced during problem solving, thereby facilitating the unprompted and appropriate use of diagrams, as well as freeing up cognitive resources that can be used in working out the answers. Evidence suggesting this was obtained in the current study: in all three problem types, instruction led to immediate or subsequent reductions in reported cognitive load, which coincided with increases in both appropriate diagram use and correct answer rates. According to cognitive load theory, the acquisition of knowledge and understanding relevant to a task leads to schema construction, which in turn leads to a reduction in intrinsic cognitive load and to freeing up of resources in working memory (Sweller et al., 1998). In the case of problem solving and diagram use, prior to instruction the experience of cognitive load would likely be high, especially if the student is unsure about what to do. However, when problem-appropriate instruction is provided, the student would learn what to do and possess a schema to use for solving the problem. This means that the student’s experience of cognitive load would likely decrease (Fuchs et al., 2020). Such decreases could have arisen because of practice effects (Wesnes and Pincock, 2002), so it would be useful in future studies to obtain direct measurements of cognitive load (e.g., brain activity). In the present study, there is also evidence from the multiple baseline design that no significant decreases in cognitive load occurred prior to instruction, even in the Compare trajectories problems with three baseline points.

### Theoretical implications

The findings of this research provide useful insights about the use of self-constructed diagrams in problem solving. They emphasize the importance of paying sufficient attention to the cultivation of procedural and conditional knowledge. In most Japanese mathematics classrooms, for example, teachers only demonstrate the use of diagrams, without any explicit explanation of how to select, construct, and use them (Uesaka et al., 2007). Despite being familiar with the types of word problems and diagrams, students did not spontaneously use diagrams and failed to solve the problems at baseline. Thus, without proper explanations students have gaps in their procedural and conditional knowledge for diagram use, which not only explain the lack of spontaneous use, but also of inappropriate use and inability to draw the necessary inferences (Hegarty and Kozhevnikov, 1999; Uesaka and Manalo, 2006; Corter and Zahner, 2007; Uesaka et al., 2007). The cultivation of the necessary (and presumably incomplete) procedural knowledge and conditional knowledge was addressed in this study through explicit instruction in problem-appropriate diagram use – which proved effective in improving problem solving behaviours.

The findings also indicate that an important consequence of such instruction is the reduction of cognitive load, specific to the problem type dealt with in the instruction. Our results suggest that cognitive load reduction is instrumental not only in promoting spontaneity in diagram use, but also in allowing sufficient cognitive resources to bear on the problem and hence to solve it successfully.

Furthermore, the findings draw attention to the distinction between two important functions that diagrams can serve in mathematical word problem solving: providing an accurate visual-schematic representation for understanding the problem and providing a schema or operational tool for solving it. Most students are aware of the first of these functions, which is why even prior to instruction many of the participants in the present study produced illustrations. However, such illustrations even when they portray an accurate schema of the problem situation, may not help in working out the solution to the problem. More complex problems often require the use of more abstract diagrams (tables, graphs) that do not visually portray the problem situation but instead directly facilitate obtaining the required solutions. In the research area of diagram use in mathematical word problem solving, little work has been undertaken on this second function (Verschaffel et al., 2020). We believe it deserves more attention as, among other things, the transformational steps involved in their construction need to be better understood.

### Research implications

In this research, the multiple baseline design allowed within-participant comparisons without requiring a control group. Multiple testing phases showed increases in performance only for the expected types of problems directly following the corresponding instruction. This design is more commonly used for evaluating individual behavioural change in response to an intervention, particularly when there is an expectation that the change would be irreversible (Baer et al., 1968; Morgan and Morgan, 2009). Apart from across individuals (participants or clients), variations of the multiple baseline design include across settings, behaviours (Morgan and Morgan, 2009), and populations (Hawkins et al., 2007). The design has previously been used to evaluate the effect of providing instruction on mathematics skills to students. However, usually, instruction in a single mathematical operations using a particular teaching approach is evaluated across individual students (Rivera and Smith, 1988). In the present study, we used the design to evaluate the effect of instruction on multiple aspects of participant responding (behaviour, performance, perception) across variations in types of problems, with the aim of demonstrating the need for problem-appropriate diagram instruction.

Like in previous studies, we expected resulting changes to be irreversible, and thus to maintain in post-instruction test phases. But we also expected the effects to be problem type-specific, with limited or no transfer across the problem types. Our results confirmed these expectations. In fact, the multiple baseline design has proven crucial in demonstrating not only the problem type-specific effects of the instructions, but also the co-occurrence of pertinent changes in behaviour, performance, and perception (increases in appropriate diagram use and correct answer rates, along with decreases in cognitive load). Therefore, from a research design perspective, we have been able to demonstrate a useful variation of the multiple baseline design that may have potential further applications in classroom educational research.

### Practical implications

The results of the present study indicate that teachers need to explicitly provide instruction on diagram use if their students are to use them effectively in mathematical word problem solving. Many students will not likely construct a diagram if they lack adequate knowledge and skills: it may seem too demanding, and any effort in constructing a diagram may not pay off. Necessary problem-appropriate diagram instruction largely depends on teachers possessing the corresponding knowledge and skills. However, some teachers may be proficient in using diagrams in mathematical word problem solving, but may not have considered how to articulate such knowledge to convey it effectively to their students. It is therefore important to incorporate training in this area both for pre-service teachers in mathematics education, as well as for in-service teachers who may need upskilling through professional development courses.

### Limitations and directions for future research

In the present study, we tested our hypotheses on the use of only three kinds of diagrams to solve three kinds of mathematical word problems. This is an important limitation to note as there are other kinds of diagrams that can be used to solve other types of problems, and it would be imperative to examine those in future research. Furthermore, our student participants all came from the same grade level in one school. We acknowledge that student capabilities in both mathematical word problem solving and diagram use would vary according to their age and grade level, as well as other aspects of their educational experiences. Thus it would also be useful to evaluate the effectiveness of problem-appropriate instruction on diagram use on students at other grade levels and from different educational backgrounds.

The instructions were also provided by the first author and an assisting teacher rather than the students’ real classroom teachers. An important step to take in future research would be to develop and evaluate instruction that real classroom teachers could use in cultivating diagram use capabilities in their own students.

## Conclusion

The results of this study indicate that instruction on diagram use enables the construction and use of appropriate diagrams, improves ability to correctly solve problems, and reduces perception of the cognitive load associated with mathematical word problem solving. The instruction needs to be problem-appropriate, meaning that students need to learn specific details about the construction and use of different kinds of diagrams relevant to solving specific types of problems. As mathematical word problem solving is one crucial means by which understanding of the relevance of mathematics in the real world is cultivated, and diagram use is arguably one of the most effective heuristics for solving them, the effect of instruction indicated by our findings warrants serious consideration – especially as the extent to which such instruction is currently provided in most classrooms may be too general and thus inadequate.

## Data availability statement

The datasets presented in this article are not readily available because the datasets generated during and/or analyzed during the current study are not publicly available because we did not obtain consent for secondary use from the participants but have a possibility to be available from the corresponding author on reasonable request. Requests to access the datasets should be directed to ayabe@academion.com.

## Ethics statement

The studies involving human participants were reviewed and approved by Psychology Research Ethics Review Board, Graduate School of Education, Kyoto University. Written informed consent to participate in this study was provided by the participants’ legal guardian/next of kin.

## Author contributions

HA and EM conceived the idea of the study. HA conducted experiments (including preliminary experiments) and drafted the original manuscript. EV developed a data scoring plan, and HA scored. EV and EM oversaw the rigor of the scoring process and its results. All authors developed the statistical analysis plan. HA conducted the analysis and EV additionally checked the results. All authors contributed to the interpretation of the results. EM supervised the conduct of this study. All authors reviewed the manuscript draft and revised it critically on intellectual content. All authors contributed to the article and approved the submitted version.

## Conflict of interest

The authors declare that the research was conducted in the absence of any commercial or financial relationships that could be construed as a potential conflict of interest.

## Publisher’s note

All claims expressed in this article are solely those of the authors and do not necessarily represent those of their affiliated organizations, or those of the publisher, the editors and the reviewers. Any product that may be evaluated in this article, or claim that may be made by its manufacturer, is not guaranteed or endorsed by the publisher.

## References

[ref1] Ainsworth S. (2006). DeFT: a conceptual framework for considering learning with multiple representations. Learn. Instr. 16, 183–198. doi: 10.1016/j.learninstruc.2006.03.001

[ref2] Ainsworth S. Th Loizou A. (2003). The effects of self-explaining when learning with text or diagrams. Cogn. Sci. 27, 669–681. doi: 10.1016/S0364-0213(03)00033-8

[ref3] Ayabe H. Manalo E. Fukuda M. Sadato N. (2021). “What diagrams are considered useful for solving mathematical word problems in Japan?” in Diagrammatic representation and inference. Diagrams 2021. Lecture notes in artificial intelligence. eds. Basu A. Stapleton G. Linker S. Legg C. Manalo E. Viana P. (Cham: Springer), 12909, 79–12983.

[ref4] Baer D. M. Wolf M. M. Risley T. R. (1968). Some current dimensions of applied behaviour analysis. J. Appl. Behav. Anal. 1, 91–97. doi: 10.1901/jaba.1968.1-91, PMID: 16795165 PMC1310980

[ref5] Boonen A. J. H. van Wesel F. Jolles J., & van der Schoot (2014). The role of visual representation type, spatial ability, and reading comprehension in word problem solving: an item-level analysis in elementary school children. Int. J. Educ. Res., 68, 15–26. doi: 10.1016/j.ijer.2014.08.001

[ref6] Butcher K. R. (2006). Learning from text with diagrams: promoting mental model development and inference generation. J. Educ. Psychol. 98, 182–197. doi: 10.1037/0022-0663.98.1.182

[ref7] Chu J. Rittle-Johnson B. Fyfe E. R. (2017). Diagrams benefit symbolic problem-solving. Br. J. Educ. Psychol. 87, 273–287. doi: 10.1111/bjep.12149, PMID: 28299771

[ref8] Cooper J. L. Sidney P. G. Alibali M. W. (2018). Who benefits from diagrams and illustrations in math problems? Ability and attitudes matter. Appl. Cogn. Psychol. 32, 24–38. doi: 10.1002/acp.3371

[ref9] Corter J. E. Zahner D. C. (2007). Use of external visual representations in probability problem solving. Stat. Educ. Res. J. 6, 22–50. doi: 10.52041/serj.v6i1.492

[ref10] DiSessa A. A. (2004). Metarepresentation: native competence and targets for instruction. Cogn. Instr. 22, 293–331. doi: 10.1207/s1532690xci2203_2

[ref11] Duval R. (1999). “Representation, vision and visualization: cognitive functions in mathematical thinking. Basic issues for learning.” in *Twenty First Annual Meeting of the North American Chapter of the International Group for the Psychology of Mathematics Education, 1, 3–26*. Available at: http://eric.ed.gov/ERICWebPortal/recordDetail?accno=ED466379%5Cnhttp://informahealthcare.com/doi/abs/10.1076/noph.25.1.3.7140

[ref12] Duval R. (2006). A cognitive analysis of problems of comprehension in a learning of mathematics. Educ. Stud. Math. 61, 103–131. doi: 10.1007/s10649-006-0400-z

[ref13] Faul F. Erdfelder E. Lang A.-G. Buchner A. (2007). G*power 3: a flexible statistical power analysis program for the social, behavioral, and biomedical sciences. Behav. Res. Methods 39, 175–191. doi: 10.3758/BF03193146, PMID: 17695343

[ref14] Fuchs L. Fuchs D. Seethaler P. M. Barnes M. A. (2020). Addressing the role of working memory in mathematical word-problem solving when designing intervention for struggling learners. ZDM-Math. Edu. 52, 87–96. doi: 10.1007/s11858-019-01070-8

[ref15] Garner R. (1990). When children and adults do not use learning strategies: toward a theory of settings. Rev. Educ. Res. 60, 517–529. doi: 10.3102/00346543060004517

[ref16] Grawemeyer B. Cox R. (2008). “The effects of users’ background diagram knowledge and task characteristics upon information display selection,” in Diagrams 2008, lecture notes in artificial intelligence. eds. Stapleton G. Howse J. Lee J., vol. 5223 (Berlin: Springer), 321–334.

[ref17] Hawkins N. G. Sanson-Fisher R. W. Shakeshaft A. D’Este C. Green L. W. (2007). The multiple baseline design for evaluating population-based research. Am. J. Prev. Med. 33, 162–168. doi: 10.1016/j.amepre.2007.03.020, PMID: 17673105

[ref18] Hegarty M. Kozhevnikov M. (1999). Types of visual-spatial representations and mathematical problem solving. J. Educ. Psychol. 91, 684–689. doi: 10.1037/0022-0663.91.4.684

[ref19] Hegarty M. Mayer R. E. Monk C. A. (1995). Comprehension of arithmetic word problems: a comparison of successful and unsuccessful problem solvers. J. Educ. Psychol. 87, 18–32. doi: 10.1037/0022-0663.87.1.18

[ref20] Hembree R. (1992). Experiments and relational studies in problem solving: a meta-analysis. J. Res. Math. Educ. 23, 242–273. doi: 10.2307/749120

[ref21] Hurley S. M. Novick L. R. (2010). Solving problems using matrix, network, and hierarchy diagrams: the consequences of violating construction conventions. Q. J. Exp. Psychol. 63, 275–290. doi: 10.1080/17470210902888908, PMID: 19440931

[ref22] Jitendra A. K. Griffin C. C. Haria P. Leh J. Adams A. Kaduvettoor A. (2007). A comparison of single and multiple strategy instruction on third-grade students’ mathematical problem solving. J. Educ. Psychol. 99, 115–127. doi: 10.1037/0022-0663.99.1.115

[ref23] Kintsch W. Greeno J. G. (1985). Understanding and solving word arithmetic problems. Psychol. Rev. 92, 109–129. doi: 10.1037/0033-295X.92.1.109, PMID: 3983303

[ref24] Leppink J. Paas F. van Gog T. van der Vleuten C. P. M. van Merriënboer J. J. G. (2014). Effects of pairs of problems and examples on task performance and different types of cognitive load. Learn. Instr. 30, 32–42. doi: 10.1016/j.learninstruc.2013.12.001

[ref25] Lewis A. B. Mayer R. E. (1987). Students’ miscomprehension of relational statements in arithmetic word problems. J. Educ. Psychol. 79, 363–371. doi: 10.1037/0022-0663.79.4.363

[ref26] Manalo E. Uesaka Y. Chen O. Ayabe H. (2019). “Showing what it looks like: teaching students how to use diagrams in problem solving, communication, and thinking,” in Deeper learning, dialogic learning, and critical thinking: Research-based strategies for the classroom. ed. Manalo E. (London: Routledge), 231–246.

[ref27] Mayer R. E. Lewis A. B. Hegarty M. (1992). “Mathematical misunderstandings: qualitative reasoning about quantitative problems,” in The nature and origins of mathematical skills. ed. Campbell J. I. D. (Amsterdam: Elsevier), 137–154.

[ref28] Morgan D. L. Morgan R. K. (2009). Single-case research methods for the behavioral and health sciences. Thousand Oaks, CA: Sage.

[ref29] Murata A. (2008). Mathematics teaching and learning as a mediating process: the case of tape diagrams. Math. Think. Learn. 10, 374–406. doi: 10.1080/10986060802291642

[ref30] Nistal A. A. van Dooren W. Clarebout G. Elen J. Verschaffel L. (2009). Conceptualising, investigating and stimulating representational flexibility in mathematical problem solving and learning: a critical review. ZDM - Int. J. Math. Educ. 41, 627–636. doi: 10.1007/s11858-009-0189-1

[ref31] Novick L. R. Hurley S. M. (2001). To matrix, network, or hierarchy: that is the question. Cogn. Psychol. 42, 158–216. doi: 10.1006/cogp.2000.0746 11259107

[ref32] OECD (2019). Programme for international student assessment (PISA): results from PISA 2018, country note: Japan. Avalailbe at: https://www.oecd.org/pisa/publications/PISA2018_CN_JPN.pdf (Accessed February 12, 2021).

[ref33] Paris S. G. Lipson M. Y. Wixson K. K. (1983). Becoming a strategic reader. Cont. Edu. Psychol. 8, 293–316. doi: 10.1016/0361-476X(83)90018-8

[ref34] Reed S. K. (1999). Word problems: Research and curriculum reform. Mahwah, NJ: Lawrence Erlbaum Associates Publishers.

[ref35] Rivera D. Smith D. D. (1988). Using a demonstration strategy to teach midschool students with learning disabilities how to compute long division. J. Learn. Disabil. 21, 77–81. doi: 10.1177/002221948802100203, PMID: 3346610

[ref36] Schmider E. Ziegler M. Danay E. Beyer L. Bühner M. (2010). Is it really robust?: reinvestigating the robustness of ANOVA against violations of the normal distribution assumption. Methodology 6, 147–151. doi: 10.1027/1614-2241/a000016

[ref37] Schnotz W. Kürschner C. (2007). A reconsideration of cognitive load theory. Educ. Psychol. Rev. 19, 469–508. doi: 10.1007/s10648-007-9053-4

[ref38] Schnotz W. Kürschner C. (2008). External and internal representations in the acquisition and use of knowledge: visualization effects on mental model construction. Instr. Sci. 36, 175–190. doi: 10.1007/s11251-007-9029-2

[ref39] Schoenfeld A. H. (1985). Mathematical problem solving. Cambridge, MA: Academic Press.

[ref40] Stylianou D. A. Silver E. A. (2004). The role of visual representations in advanced mathematical problem solving: an examination of expert-novice similarities and differences. Math. Think. Learn. 6, 353–387. doi: 10.1207/s15327833mtl0604_1

[ref41] Suthers D. D. (2003). Representational guidance for collaborative inquiry. Arg. Learn 1994, 27–46. doi: 10.1007/978-94-017-0781-7_2

[ref42] Sweller J. (1994). Cognitive load theory, learning difficulty, and instructional design. Learn. Instr. 4, 295–312. doi: 10.1016/0959-4752(94)90003-5

[ref43] Sweller J. van Merrienboer J. J. G. Paas F. G. W. C. (1998). Cognitive architecture and instructional design. Educ. Psychol. Rev. 10, 251–296. doi: 10.1023/A:1022193728205

[ref44] Uesaka Y. Manalo E. (2006). “Active comparison as a means of promoting the development of abstract conditional knowledge and appropriate choice of diagrams in math word problem solving” in Diagrams 2006. Lecture notes in artificial intelligence 4045. eds. Barker-Plummer D. Cox R. Swoboda N. (Berlin: Springer), 181–195.

[ref45] Uesaka Y. Manalo E. (2012). Task-related factors that influence the spontaneous use of diagrams in math word problems. Appl. Cogn. Psychol. 26, 251–260. doi: 10.1002/acp.1816

[ref46] Uesaka Y. Manalo E. Ichikawa S. (2007). What kinds of perceptions and daily learning behaviors promote students’ use of diagrams in mathematics problem solving? Learn. Instr. 17, 322–335. doi: 10.1016/j.learninstruc.2007.02

[ref47] Uesaka Y. Manalo E. Ichikawa S. (2010). “The effects of perception of efficacy and diagram construction skills on students' spontaneous use of diagrams when solving math word problems” in Diagrams 2010. Lecture notes in artificial intelligence 6170. eds. Goel A. K. Jamnik M. Narayanan N. H. (Berlin Springer), 197–211.

[ref48] van der Meij J. van Amelsvoort M. Anjewierden A. (2017). How design guides learning from matrix diagrams. Instr. Sci. 45, 751–767. doi: 10.1007/s11251-017-9425-1

[ref49] van Garderen D. (2007). Teaching students with LD to use diagrams to solve mathematical word problems. J. Learn. Disabil. 40, 540–553. doi: 10.1177/00222194070400060501, PMID: 18064979

[ref50] van Garderen D. Scheuermann A. Jackson C. (2012). Examining how students with diverse abilities use diagrams to solve mathematics word problems. Learn. Disabil. Q. 36, 145–160. doi: 10.1177/0731948712438558

[ref51] van Meter P. Garner J. K. (2005). The promise and practice of learner-generated drawing: literature review and synthesis. Educ. Psychol. Rev. 17, 285–325. doi: 10.1007/s10648-005-8136-3

[ref52] Verschaffel L. Schukajlow S. Star J. Van Dooren W. (2020). Word problems in mathematics education: a survey. ZDM-Math. Edu. 52, 1–16. doi: 10.1007/s11858-020-01130-4

[ref53] Wesnes K. Pincock C. (2002). Practice effects on cognitive tasks: a major problem? Lancet Neurol. 1:473. doi: 10.1016/S1474-4422(02)00236-3, PMID: 12849328

[ref54] Zahner D. Corter J. E. (2010). The process of probability problem solving: use of external visual representations. Math. Think. Learn. 12, 177–204. doi: 10.1080/10986061003654240

[ref55] Zhang J. (1997). The nature of external representations in problem solving. Cogn. Sci. 21, 179–217. doi: 10.1016/S0364-0213(99)80022-6

[ref56] Zhang J. Norman D. A. (1994). Representations in distributed cognitive tasks. Cogn. Sci. 18, 87–122. doi: 10.1207/s15516709cog1801_3

